# Rectal Gland Chemistry, Volatile Emissions, and Antennal Responses of Male and Female Banana Fruit Fly, *Bactrocera musae*

**DOI:** 10.3390/insects11010032

**Published:** 2019-12-31

**Authors:** Saeedeh Noushini, Jeanneth Perez, Soo Jean Park, Danielle Holgate, Ian Jamie, Joanne Jamie, Phillip Taylor

**Affiliations:** 1 Department of Molecular Sciences, Macquarie University, Sydney NSW 2109, Australia; 2 Australian Research Council Industrial Transformation Training Centre for Fruit Fly Biosecurity Innovation, Macquarie University, Sydney NSW 2109, Australia; 3 Applied BioSciences, Macquarie University, Sydney NSW 2109, Australia

**Keywords:** *B. musae*, headspace, electroantennography, insect volatile, GC-EAD

## Abstract

The banana fruit fly, *Bactrocera musae* (Tryon) (Diptera: Tephritidae), is an economically important pest endemic to Australia and mainland Papua New Guinea. The chemistry of its rectal glands, and the volatiles emitted during periods of sexual activity, has not been previously reported. Using gas chromatography–mass spectrometry (GC-MS), we find that male rectal glands contain ethyl butanoate, *N*-(3-methylbutyl) acetamide, ethyl laurate and ethyl myristate, with ethyl butanoate as the major compound in both rectal gland and headspace volatile emissions. Female rectal glands contain four major compounds, ethyl laurate, ethyl myristate, ethyl palmitate and (*E*,*E*)-2,8-dimethyl-1,7-dioxaspiro[5.5]undecane, as well as 11 minor compounds. For both male and female *B.* *musae*, all compounds found in the headspace were also present in the rectal gland extracts, suggesting that the rectal gland is the main source of the headspace volatiles. Gas chromatography–electroantennography (GC-EAD) of rectal gland extracts confirms that male antennae respond to male-produced ethyl laurate and female-produced (*E*,*E*)-2,8-dimethyl-1,7-dioxaspiro[5.5]undecane, while female antennae respond to male-produced ethyl butanoate but no female-produced compounds. This is an important step in understanding the volatiles involved in the chemical communication of *B. musae*, their functional significance, and potential application.

## 1. Introduction

In tephritid fruit flies (Diptera: Tephritidae), courtship and mating are typically mediated by pheromones [1,2]. Sex pheromones are usually secreted and stored in the rectal glands and emitted during periods of mating activity [3,4,5,6]. The volatile compounds released by fruit flies are known to attract the opposite sex in many species [7,8,9,10], as well as members of the same sex to form mating aggregations [11,12]. Although males are commonly thought of as the major sex pheromone producers [13,14], there are also examples of female fruit flies that produce and release sex pheromones. For example, in *Bactrocera oleae* (Rossi), sex pheromones are produced mainly by the female [15,16], while for *Zeugodacus cucurbitae* (Coquillett) and *Bactrocera dorsalis* (Hendel), both male and female volatile emissions attract the opposite sex [17,18,19,20].

*Bactrocera musae* (Tryon) is a polyphagous pest species endemic to Australia and mainland Papua New Guinea [21]. In Australia, *B. musae* is very common along the eastern coast of Queensland as far south as Townsville [22], where bananas (*Musa* spp.) are an important commercial crop [23]. Unlike many other fruit fly species that oviposit in ripe fruits, *B. musae* lays eggs in unripe bananas [24,25]. Therefore, harvesting bananas at a green stage to avoid fruit fly infestation is not a solution in these regions [24]. Although banana is its major host, papaya (*Carica papaya* L.) and guava (*Psidium guajava* L.) are also occasional hosts of *B. musae* [23]. Sex pheromones of many of the most economically important *Bactrocera* species have been documented [2,15,17,18,26,27,28,29,30]. However, the chemical profiles of rectal gland and volatile emissions of *B. musae* are unknown, despite its moderate to high pest status [31]. This study presents the rectal gland chemical profiles of both male and female *B. musae*, as well as headspace samples, using gas chromatography–mass spectrometry (GC-MS), and evaluates the antennal electrophysiological response of male and female *B. musae* to the volatiles produced by the opposite sex using gas chromatography–electroantennogram detection (GC-EAD).

## 2. Materials and Methods

### 2.1. Banana Fruit Fly Rearing

Laboratory-reared populations of *B. musae* were obtained from the Queensland Government Department of Agriculture and Fisheries (QDAF) in September 2014 (Mareeba) and November 2017 (Cairns).

2014 collections: Flies were kept in mixed-sex cages at QDAF, Mareeba, in a controlled environment room at 26 ± 1 °C, 70 ± 5% relative humidity (RH) and with a natural light cycle so the flies experienced a natural dusk. The adult flies were fed with sugar and yeast hydrolysate provided separately, and water through a soaked sponge. Flies used for rectal gland extractions were 13–18 days old.

2017 collections: Flies were kept at Macquarie University, Sydney, in a controlled environment room at 25 ± 0.5 °C, 65 ± 5% RH and 11.5:0.5:11.5:0.5 light/dusk/dark/dawn photoperiod. The adult flies were fed with sugar and yeast hydrolysate (MP Biomedicals LLC, Santa Ana, CA, USA) provided separately, and water through a soaked sponge. Flies that had been received as pupae were reared through one generation using a standard carrot diet [32], following the method described by Pérez et al. [33]. Flies were sorted by sex within three days after emergence and transferred to 12.5 L clear plastic cages (180 flies per cage). No mating was observed before separating the flies. Flies used for all experiments were 13–18 days old.

### 2.2. Rectal Gland Extractions

*n-*Hexane extracts of rectal glands of flies collected in both 2014 and 2017 were obtained using sexually mature male and female *B. musae* (13–18 days old) following literature procedures [34]. Flies were chilled on dry ice and then dissected. The abdomen was gently squeezed with tweezers such that the gland protruded slightly, and the gland was gently pulled out with fine forceps and carefully placed in a tear-drop vial (1.1 mL) in dry ice. For each sex and for each year of collection, 20 glands were combined in the vials. The vials were taken out of the dry ice, and 200 μL of *n*-hexane (HPLC grade, Sigma-Aldrich, St. Louis, MO, USA) was added to each vial. Samples were left to stand at room temperature for 10 min, then the extracts were transferred to a new labelled vial and stored at −20 °C until analysed. Three replicates per sex were collected in 2014, and six replicates were collected in 2017.

### 2.3. Headspace Collections

Collections of volatile emissions from male and female *B. musae* obtained from QDAF in 2017 were performed at Macquarie University, Sydney, in a controlled environment room at 25 ± 0.5 °C and 65 ± 5% RH. Like many other *Bactrocera*, *B. musae* mate at dusk [35]. Thirty sexually mature males and 30 sexually mature females (13–18 days old) were separately placed into a cylindrical glass chamber (150 mm long and 40 mm inner diameter) 30 min before dusk. A charcoal-filtered air stream at a flow rate of 0.5 L/min (air pulling system) was drawn over the flies for one hour, from the beginning of dusk. Released volatiles were adsorbed onto traps of 50 mg of Tenax-GR adsorbent (Scientific Instrument Services, Inc, Ringoes, NJ, USA, Tenax-GR Mesh 60/80) packed into 6 × 50 mm glass tubes and fitted with glass wool plugs. Volatiles were eluted with 1 mL of *n-*hexane (HPLC grade, Sigma-Aldrich). An air control sample comprising an empty glass chamber was run and analysed along with each volatile collection. Samples were stored at −20 °C until analysis. Six replicates of 30 flies per sex were obtained. Prior to each headspace collection, Tenax traps were conditioned at 200 °C for three hours under a nitrogen stream (75 mL/min). Glass chambers were washed with 5% Extran aqueous solution, rinsed with hot tap water, and heated at 200 °C for 18 h. Activated charcoal filters were thermally conditioned by heating them at 200 °C for 18 h prior to each headspace collection [36].

### 2.4. GC-MS Analysis

Mass spectra were recorded on a Shimadzu GCMS-QP2010 instrument. The GC was equipped with a non-polar capillary column with 5% diphenyl/95% dimethyl polysiloxane as the stationary phase (SH-Rtx-5MS, 30 m × 0.25 mm ID × 0.25 μm film thickness, Shimadzu, Japan) and helium (99.999%) (ultra-high purity, BOC, Australia) as a carrier gas with a constant flow of 1 mL/min. A 1 μL sample was injected in the splitless mode. The injector temperature was set at 270 °C. The temperature program was 50 °C (4 min) to 250 °C (6 min) at a rate of 10 °C/min. The interphase and ion source temperatures were set at 290 °C and 200 °C, respectively. Mass spectra were recorded in electron impact mode (70 eV), scanning from 40 to 620 *m*/*z*. A peak was considered of interest if it was not present in the air control samples. The identification of compounds was confirmed by comparing their mass spectra and retention times to those of commercial standards or synthesised samples. Commercial samples were purchased from Sigma-Aldrich (Castle Hill, Australia), Alfa-Aesar (Heysham, Lancashire, United Kingdom) and Nu-Chek-Prep, INC (Minneapolis, MN, USA). These compounds included ethyl butanoate, ethyl caprate, methyl laurate, ethyl laurate, ethyl tridecanaote, methyl myristate, ethyl myristate, ethyl myristoleate, methyl palmitate, ethyl palmitate and ethyl oleate. (*E*,*E*)-2,8-Dimethyl-1,7-dioxaspiro[5.5]undecane (racemate), *N*-(3-methylbutyl)acetamide, propyl laurate, ethyl palmitoleate and ethyl elaidate were not commercially available and were synthesised following literature procedures (see Supplementary Materials for synthesis details). (*E*,*E*)-2-Ethyl-8-methyl-1,7-dioxaspiro[5.5]undecane was tentatively identified based on the literature mass spectral fragmentation pattern [37].

### 2.5. GC-EAD Experiments

Gas chromatography–electroantennographic detection (GC-EAD) was conducted using a gas chromatography flame ionisation detector (FID, Agilent 7890B) coupled to an electroantennogram (Ockenfels Syntech GmbH, Kirchzarten, Germany). The GC was equipped with a non-polar capillary column with (5%-phenyl)-methylpolysiloxane as the stationary phase (Agilent HP-5, 30 m × 0.32 mm ID × 0.25 μm film thickness). The carrier gas was hydrogen (99.999% pure) supplied by a generator (MGG-2500-220 Parker Balston, NY, USA) with a constant flow of 2.5 mL/min. The initial temperature was set at 50 °C (4 min) then increased to 250 °C (6 min) at a rate of 10 °C/min. The injector and detector temperatures were set at 270 °C and 290 °C, respectively. The effluent of the column was mixed with 30 mL/min make-up nitrogen gas and split in a ratio of 1 (FID) to 1.5 (EAD) through a heated transfer line (Syntech, TC-02, Ockenfels Syntech GmbH, Kirchzarten, Germany) and kept at 200 °C.

A female or male B. musae head was carefully severed and a silver glass capillary electrode filled with phosphate-buffered saline (PBS) was inserted into the back of the head. The tip of the antenna was inserted into the tip of the recording glass capillary electrode. The mounted heads were placed under a charcoal-filtered and humidified air flow (400 mL/min) controlled by a flow controller (Syntech Stimulus Controller CS-55, Ockenfels Syntech GmbH, Kirchzarten, Germany) and were subjected to each stimulus. Electrophysiological responses were captured and processed by a data acquisition controller (IDAC-4, Ockenfels Syntech GmbH, Kirchzarten, Germany). Before the injection of the sample into the airstream, the antenna was stimulated with 1-hexanol to check its sensitivity; then, 1 µL of the rectal gland extract was injected. EAD signals were analysed using GC-EAD 2014 software version 1.2.5. Nine successful GC-EAD recordings were obtained for each sex. The electrophysiological responses of male and female antennae to the conspecific opposite sex rectal glands extract were recorded. A response was considered genuine if it was present in at least six out of the nine replicates collected. The identity of the compounds eliciting an electrophysiological response was confirmed by comparing retention times with those of GC-MS chromatograms.

## 3. Results

### 3.1. Analysis of Volatile Compounds

**Figure 1:**
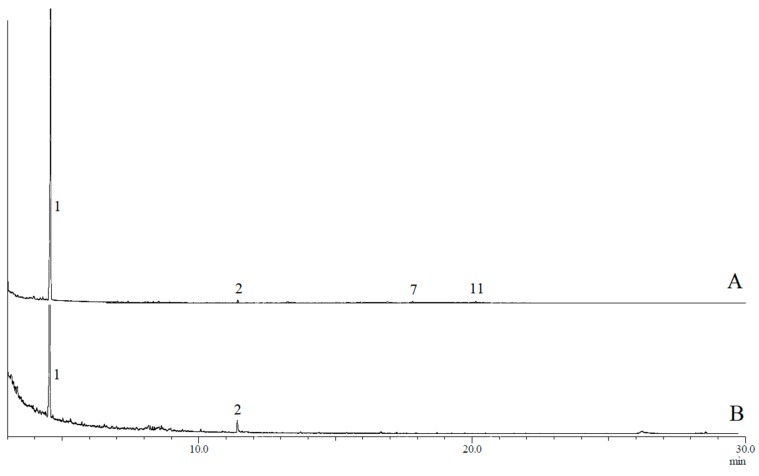
Typical gas chromatogram of (**A**) rectal gland extract and (**B**) headspace collections of *B. musae* males. Numbered peaks indicate detected compounds: ethyl butanoate (**1**), *N*-(3-methylbutyl)acetamide (**2**), ethyl laurate (**7**) and ethyl myristate (**11**).

**Figure 2:**
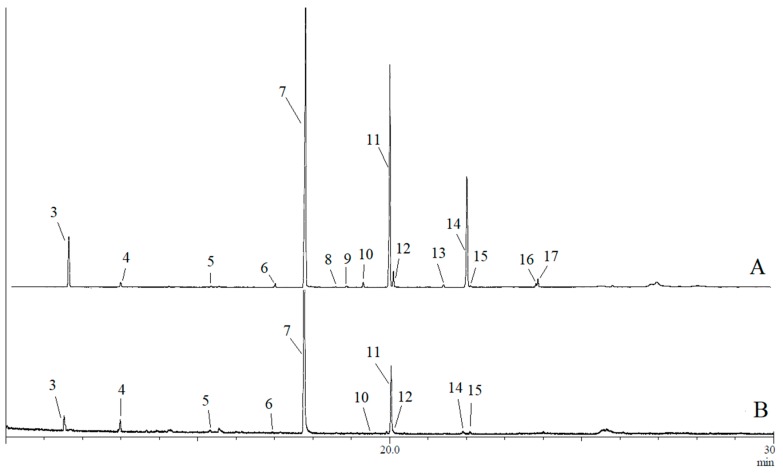
Typical gas chromatogram of (**A**) rectal gland extract and (**B**) headspace collections of *B. musae* females. Numbered peaks indicate detected compounds: (*E*,*E*)-2,8-dimethyl-1,7-dioxaspiro[5.5]undecane (**3**), (*E*,*E*)-2-ethyl-8-methyl-1,7-dioxaspiro[5.5]undecane (**4**), ethyl caprate (**5**), methyl laurate (**6**), ethyl laurate (**7**), ethyl tridecanaote (**8**), propyl laurate (**9**), methyl myristate (**10**), ethyl myristate (**11**), ethyl myristoleate (**12**), methyl palmitate (**13**), ethyl palmitate (**14**), ethyl palmitoleate (**15**), ethyl oleate (**16**) and ethyl elaidate (**17**).

GC-MS analyses confirmed the presence of 17 compounds in rectal gland extracts and headspace collections of sexually mature male and female *B. musae* (Table 1). This included 14 esters, one amide and two spiroacetals. For both males and females, a more complex blend was detected in the rectal glands, with fewer compounds detected for the headspace collections for both sexes (Figure 1 and Figure 2). No additional compounds were detected in the headspace samples. The most abundant chemicals in female rectal glands, (*E*,*E*)-2,8-dimethyl-1,7-dioxaspiro[5.5]undecane (**3**), (*E*,*E*)-2-ethyl-8-methyl-1,7-dioxaspiro[5.5]undecane (**4**), ethyl caprate (**5**), methyl laurate (**6**), ethyl laurate (**7**), methyl myristate (**10**), ethyl myristate (**11**), ethyl myristoleate (**12**), ethyl palmitate (**14**) and ethyl palmitoleate (**15**) were also detected in the headspace collections. Similarly, ethyl butanoate (**1**) and *N-*(3-methylbutyl)acetamide (**2**) were found in both the male rectal glands and headspace samples. Overall, there was a large difference in the chemical profiles of males and females. Males predominantly produced and released a short carbon chain ester, ethyl butanoate (**1**). Females predominantly produced and released longer carbon chain esters, ethyl laurate (**7**) and ethyl myristate (**11**).

### 3.2. Assignment of Compounds

Amide: Compound **2** was found to have an odd molecular ion at *m*/*z* 129, indicating the presence of a single nitrogen atom in the molecule, a fragment ion at *m*/*z* 114 due to loss of a methyl group, and an *m*/*z* 60 fragment ion consistent with β-cleavage of acetamides [38]. It also showed a fragment ion at *m*/*z* 86, consistent with the loss of a C_3_H_7_ or COCH_3_ moiety. The NIST library showed high similarity for compound **2** with *N*-(3-methylbutyl)acetamide, and this identity was confirmed by comparing the retention time and mass spectral fragmentation pattern of the compound in the rectal gland extracts and headspace collections with the synthesized amide.

Methyl esters: Compounds **6**, **10** and **13** all produced a base peak at *m*/*z* 74, which is characteristic of the McLafferty rearrangement of methyl esters [39]. Compounds **6**, **10** and **13** exhibited molecular ions at *m*/*z* 214, *m*/*z* 242 and *m*/*z* 270, respectively, and they all showed loss of *m*/*z* 31 consistent with cleavage of a methoxy group, suggesting they were methyl esters of saturated C_12_, C_14_ and C_16_ chains, respectively. A NIST library search showed high similarity with methyl laurate, methyl myristate and methyl palmitate for compounds **6**, **10** and **13**, respectively. The identities of the compounds were confirmed by comparing retention times and mass spectral fragmentation patterns of the compounds in the rectal gland extracts and headspace collections with the authentic commercial methyl esters.

Ethyl esters: Compounds **5**, **7**, **8**, **11** and **14** exhibited molecular ions at *m*/*z* 200, *m*/*z* 228, *m*/*z* 242, *m*/*z* 256 and *m*/*z* 284, respectively, along with the characteristic McLafferty fragmentation product of ethyl esters at *m*/*z* 88 as a base peak [39]. They also all showed similar fragmentation patterns, including fragment ions from the cleavage of an ethyl group, ethoxy group, and propyl group from the molecular ion. These data indicated compounds **5**, **7**, **8**, **11** and **14** were ethyl esters of saturated C_10_, C_12_, C_14_, C_15_ and C_16_ chains, respectively. Compounds **12**, **15**, **16** and **17** had molecular ions at *m*/*z* 254, *m*/*z* 282 and *m*/*z* 310, respectively. They all showed a loss of ethanol from the molecular ion, and the McLafferty fragment ion at *m*/*z* 88 was present but less abundant than for the saturated esters **5**, **7**, **8**, **11** and **14**. A NIST library search suggested compounds **5**, **7**, **8**, **11** and **14** were the saturated esters ethyl caprate, ethyl laurate, ethyl tridecanaote, ethyl myristate and ethyl palmitate and **12**, **15**, **16** and **17** were the unsaturated esters ethyl myristoleate, ethyl palmitoleate, ethyl oleate and ethyl elaidate, respectively. The identities of the esters were confirmed by comparing retention times and mass spectral fragmentation patterns of the compounds in the rectal gland extracts and headspace collections with the authentic commercial or synthesized samples.

Propyl ester: Compound **9** showed a molecular ion at *m*/*z* 242 and a McLafferty fragment ion at *m*/*z* 102, suggesting a propyl/isopropyl ester with a C_12_ saturated chain [39]. It also exhibited a fragment ion at *m*/*z* 183 for loss of a propoxy (C_3_H_7_O) group. Given that propyl and isopropyl esters are structural isomers and will therefore produce very similar mass spectra, both propyl laurate and isopropyl laurate were synthesized. Compound **9** was found to have very similar mass spectral fragmentation patterns to the propyl isomer (but not isopropyl laurate). The identification of compound **9** was further confirmed as propyl laurate based on a comparison of retention time by the co-injection of the rectal gland extracts and synthesized propyl laurate. Isopropyl laurate showed a slightly different retention time when compared to compound **9**.

Spiroacetals: Compounds **3** and **4** had molecular ions of *m*/*z* 184 and 198, respectively, and similar mass spectral fragmentation patterns, indicating a difference of CH_2_ only. This was further supported by both compounds exhibiting a fragment ion at *m*/*z* 169, indicating a loss of CH_3_ for compound **3** and CH_2_CH_3_ for compound **4**. Both compounds also showed characteristic fragmentation patterns of methyl substituted dioxaspiro[5.5]undecane spiroacetals [40], including a strong doublet at *m*/*z* 112 and *m*/*z* 115, due to the retro-cleavage of one of the six-membered rings leading to a methyl methylene heterocycle (CH_3_(C_5_H_7_O)=CH_2_) at *m*/*z* 112 and *m*/*z* 115 (CH_3_(C_5_H_7_O)=OH) due to the alternate ring cleavage accompanied by intramolecular hydrogen transfer. The presence of another set of ions at *m*/*z* 126 and *m*/*z* 129 for compound **4** indicated it also had an ethyl substituted ring. Compound **3** was consistent with a dimethylated dioxaspiro[5.5]undecane, while compound **4** was consistent with a methyl and ethyl substituted dioxaspiro[5.5]undecane [40]. Compound **3** was confirmed as (*E*,*E*)-2,8-dimethyl-1,7-dioxaspiro[5.5]undecane by comparing the retention time and mass spectral fragmentation pattern of the compound in the rectal gland extracts and headspace collections with the authentic synthesized (racemic) sample. Compound **4** was tentatively identified as (*E*,*E*)-2-ethyl-8-methyl-1,7-dioxaspiro[5.5]undecane based on the literature mass spectral fragmentation pattern [8,37,41].

For *B. musae* males, four compounds were identified in rectal gland extracts obtained from the flies collected in 2014, including ethyl butanoate (**1**), *N*-(3-methylbutyl)acetamide (**2**), ethyl laurate (**7**) and ethyl myristate (**11**), while only compounds **1** and **2** were detected for the 2017 collections. Of the four compounds, only ethyl butanoate (**1**) and *N*-(3-methylbutyl)acetamide (**2**) were found in the headspace samples. In contrast, for *B. musae* females, 15 compounds were identified in rectal gland extracts obtained from flies collected in both 2014 and 2017 (Table 1). Identified compounds included 13 saturated/unsaturated fatty acid esters, including ethyl caprate (**5**), methyl laurate (**6**), ethyl laurate (**7**), ethyl tridecanaote (**8**), propyl laurate (**9**), methyl myristate (**10**), ethyl myristate (**11**), ethyl myristoleate (**12**), methyl palmitate (**13**), ethyl palmitate (**14**), ethyl palmitoleate (**15**), ethyl oleate (**16**) and ethyl elaidate (**17**), as well as two spiroacetals (*E*,*E*)-2,8-dimethyl-1,7-dioxaspiro[5.5]undecane (**3**) and (*E*,*E*)-2-ethyl-8-methyl-1,7-dioxaspiro[5.5]undecane (**4**). Of these, ten compounds were also found in headspace collections. The main compound present in female gland extracts and headspace samples was ethyl laurate (**7**), although it was found in higher proportions in the headspace samples (~73% vs. 47%).

### 3.3. GC-EAD Experiment

**Figure 3:**
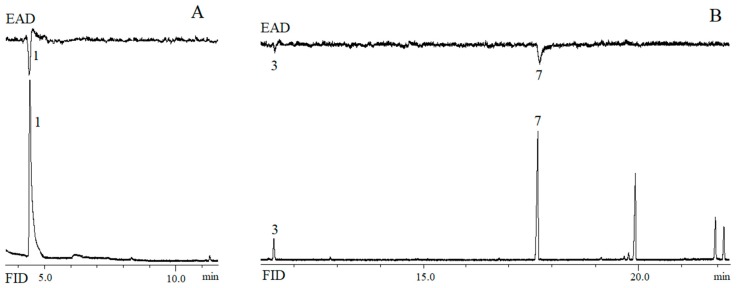
Simultaneous response of flame ionisation detector (FID) and electroantennographic detection (EAD) using *B. musae* (**A**) female antenna with rectal gland extract from conspecific males and (**B**) male antenna to rectal gland extract from conspecific females. Numbered peaks indicate EAD-active compounds: ethyl butanoate (**1**), (*E*,*E*)-2,8-dimethyl-1,7-dioxaspiro[5.5]undecane (**3**) and ethyl laurate (**7**).

The electroantennogram response of female and male *B. musae* to the rectal gland extract of the conspecific opposite sex is shown in Figure 3. Ethyl butanoate (**1**), the most abundant compound emitted by male *B. musae*, elicited antennal responses from *B. musae* females. Two compounds, (*E*,*E*)-2,8-dimethyl-1,7-dioxaspiro[5.5]undecane (**3**) and ethyl laurate (**7**), elicited antennal responses from conspecific males.

## 4. Discussion

The present study describes for the first time the chemical profiles of rectal gland extracts and volatiles released during dusk, the period of sexual activity in *B. musae*, as well as the EAD-active compounds for both sexes. Females are found to release a more complex blend than males, and the major compound(s) present in the chemical profile of each sex elicited antennal responses in the opposite sex.

Female rectal gland volatile profiles from colonies collected in 2014 and 2017 were similar in composition and relative amounts. There were some differences in the male rectal gland profiles between the two collections. Male rectal glands from the flies obtained in 2014 contained trace amounts of ethyl laurate and ethyl myristate (<0.5%), which were not observed in flies collected in 2017. Qualitative and/or quantitative changes in the volatile composition may appear as a result of age, nutritional and mating status [30,42,43,44], or due to the domestication process [33]. The differences found between the two collections cannot easily be explained as larvae and adults used for both collections were fed the same diet (carrot diet for larvae and sugar and hydrolysate yeast for adults), and the adults used for the extractions were in the same age range (13–18 days old). Therefore, it is unlikely that these factors can cause this slight difference. The difference may have arisen because the samples collected in 2014 were from mixed sex cages of flies whereas the samples collected in 2017 were from single sex cages.

Ethyl butanoate (**1**), the major component found in male *B. musae* rectal gland and headspace extracts, has not been previously found in volatile secretions/emissions of other tephritid fruit fly species. It is commonly found in fruits, such as mangos, and is known to elicit EAD responses in female *B. dorsalis* [45]. *N*-(3-Methylbutyl)acetamide (**2**) has been reported in other fruit fly pheromone profiles, and elicits female attraction in *Z. cucurbitae*, *B. dorsalis* and *Bactrocera carambolae* Drew & Hancock [3,6,18]. Ethyl laurate (**7**) and ethyl myristate (**11**) were only minor compounds in males, but represent more than 90% of abundance in female *B. musae*.

Saturated/unsaturated fatty acid esters are commonly found in rectal glands of female *Bactrocera* [46]. All saturated/unsaturated fatty acid esters from female *B. musae* have been previously reported in rectal gland extracts of female *Bactrocera tryoni* (Froggatt) [46,47,48]. Ethyl myristate (**11**) and ethyl palmitate (**14**) are also found in rectal gland extracts of male *Bactrocera jarvisi* (Tryon) [47]. These two compounds, as well as methyl laurate (**6**), have also been reported as minor components in the rectal gland extracts of female *B. oleae* [49]. Ethyl laurate (**7**), the most abundant compound found in *B. musae* females, has been previously reported in rectal gland extracts of female *B. oleae* [49] and male *B. jarvisi* [47]. The relative abundance of this compound is higher in headspace than rectal glands due to its lower chain length (C_12_) and hence its higher volatility in headspace compared to the C_14_, C_16_ or C_18_ esters. Males of *B. dorsalis* exhibit EAD responses to this compound, which was found in the cuticle extraction [50]. Surprisingly, Ethyl laurate (**7**) only elicited EAD responses from *B. musae* males.

Both spiroacetals, (*E,E*)-2,8-dimethyl-1,7-dioxaspiro[5.5]undecane (**3**) and (*E*,*E*)-2-ethyl-8-methyl-1,7-dioxaspiro[5.5]undecane (**4**), have been reported as volatile emissions of fruit flies. (*E,E*)- 2,8-Dimethyl-1,7-dioxaspiro[5.5]undecane (**3**) has been found in rectal glands of many fruit flies including *B. dorsalis*, *B. nigrotibialis* (Perkins), *B. albistrigata* (Meijere), *B. jarvisi*, *B. kirki* (Froggatt), *B. kraussi* (Hardy), *Z. cucumis* and *B. tryoni* [34,46,47,48,51,52]. (*E*,*E*)-2-Ethyl-8-methyl-1,7-dioxaspiro[5.5]undecane (**4**) has been previously reported as part of male emissions of male *B. nigrotibialis*, *B. halfordiae* (Tryon), *B. dorsalis*, *B. kirki*, *B. latifrons* (Hendel) and *B. occipitalis* (Bezzi) as well as female *B. tryoni* [2,3,46,53,54]. For *B. nigrotibialis* and *Z. cucumis*, the spiroacetals were found to be solely the 2*S*,6*R*,8*S* enantiomers [34,55]. It is likely that the spiroacetals (**3**) and (**4**) found in *B. musae* also exist as single enantiomers, but this was not investigated in our study. The finding that (*E*,*E*)-2,8-dimethyl-1,7-dioxaspiro[5.5]undecane (**3**) elicited an antennal response in males suggests a likely biological role of this compound, together with ethyl butanoate (**1**) and ethyl laurate (**7**).

## 5. Conclusions

The investigation of rectal gland and airborne volatiles of *B. musae* at dusk, the period of mating activity in this species, revealed that males and females produce and release distinctly different volatile compounds. Females were found to release a more complex blend of volatile compounds than males. Males predominantly produce a short carbon chain ester—ethyl butanoate—while females predominantly produce longer carbon chain esters—ethyl laurate, ethyl myristate, ethyl palmitate. For both males and females, all compounds found in the headspace collections were also present in the rectal gland extracts. Furthermore, GC-EAD results showed that the major compound present in the chemical profile of each sex elicited an antennal response in the opposite sex, suggesting a possible biological role of these compounds in the mating system of *B. musae*. Knowing the volatiles that are released during mating activity and those that elicit antennal responses is an important step toward understanding the chemical communication system of the banana fruit fly *B. musae*. However, further behavioural studies are required in order to investigate the functions of the volatiles we identified in male and female rectal glands to conspecific females or males (e.g., attraction, species and sex identification, indicators of mate quality). Such insight into the sexual communication of this species could reveal new applications for the control of this pest.

## Figures and Tables

**Table 1:** Relative amount of compounds identified in chemical profiles for *B. musae*. KI = Kováts retention index, ND = not detected.

KI	Compound	Female			Male		
		Rectal Glands		Headspace	Rectal Glands		Headspace
		2014	2017	2017	2014	2017	2017
828	Ethyl butanoate (**1**)	ND	ND	ND	99.0	99.2	98.3
1162	*N*-(3-Methylbutyl)acetamide (**2**)	ND	ND	ND	<1	<1	1.7
1179	(*E*,*E*)-2,8-Dimethyl-1,7-dioxaspiro[5.5]undecane (**3**)	5.0	5.7	2.1	ND	ND	ND
1266	(*E*,*E*)-2-Ethyl-8-methyl-1,7-dioxaspiro[5.5]undecane (**4**)	<1	<1	2.0	ND	ND	ND
1437	Ethyl caprate (**5**)	<1	<1	<1	ND	ND	ND
1570	Methyl laurate (**6**)	<1	<1	<1	ND	ND	ND
1637	Ethyl laurate (**7**)	40.0	47.3	72.5	<1	ND	ND
1705	Ethyl tridecanaote (**8**)	<1	<1	ND	ND	ND	ND
1731	Propyl laurate (**9**)	<1	<1	ND	ND	ND	ND
1771	Methyl myristate (**10**)	<1	<1	<1	ND	ND	ND
1837	Ethyl myristate (**11**)	21.8	25.2	19.2	<1	ND	ND
1845	Ethyl myristoleate (**12**)	1.9	1.7	<1	ND	ND	ND
1974	Methyl palmitate (**13**)	<1	<1	ND	ND	ND	ND
2037	Ethyl palmitate (**14**)	21.7	16.4	1.6	ND	ND	ND
2047	Ethyl palmitoleate (**15**)	<1	<1	<1	ND	ND	ND
2233	Ethyl oleate (**16**)	<1	<1	ND	ND	ND	ND
2239	Ethyl elaidate (**17**)	<1	<1	ND	ND	ND	ND

## Supplementary Materials

The following are available online at https://www.mdpi.com/2075-4450/11/1/32/s1: synthesis of *N*-(3-methylbutyl)acetamide (**2**), 2,8-dimethyl-1,7-dioxaspiro[5.5]undecane (**3**), propyl laurate (**9**), isopropyl laurate, ethyl palmitoleate (**15**) and ethyl elaidate (**17**).
